# Preparation and characterization of an algal-based magnetic biochar nanocomposite for the removal of azocarmine G2 dye from aqueous solutions

**DOI:** 10.1186/s13065-025-01474-6

**Published:** 2025-05-11

**Authors:** Mohamed Saad Hellal, Sayed K. Attia, Kishore Kumar Kadimpati, Anna Gnida, Ahmed M. Rashad

**Affiliations:** 1 Water Pollution Research Department, National Research Centre, Cairo, 12622 Egypt; 2 Analysis and Evaluation Department, Egyptian Petroleum Research Institute, Nasr City, Cairo Egypt; 3 Department of Environmental Biotechnology, Faculty of Environmental and Energy Engineering, Silesian University of Technology, 44-100 Gliwice, Poland; 4 Department of Pharmaceutics, Swathi College of Pharmacy, Venkatachalam, Nellore, Andhra Pradesh 524 320 India

**Keywords:** Magnetic biochar, Azocarmine G2, Adsorption, Kinetics, Nanocomposite, Wastewater

## Abstract

Dyes are released into bodies of water as the textile industry expands in response to the growth of the global population. These textile dyes have severe effects on the environment, including wildlife, terrestrial species, and humans. This study explores the synthesis, characterization, and application of an algal-based magnetic biochar nanocomposite for the efficient adsorption of azocarmine G2 (ACG2) dye from aqueous solutions. The magnetic biochar (Fe_3_O_4_@BC) was characterized by Raman spectroscopy, scanning electron microscopy (SEM), X-ray diffraction (XRD), and Fourier transform infrared spectrometry (FTIR). Batch adsorption experiments were performed to assess the impact of the initial dye concentration (25 to 100 mg / L), contact time (up to 300 min), pH (1–3) and temperature (298 to 328 K). The nano-composite achieved a maximum adsorption capacity (q_max_) of 71.3 mg/g at pH 1, with equilibrium reached within 240 min. Adsorption kinetics followed a pseudo-second-order model (R2 = 0.99), while isotherm analysis fit well with the Langmuir model (R2 = 0.98), indicating monolayer adsorption. However, the Freundlich model provided a better fit, indicating that the multilayer covered a heterogeneous surface with a chemisorption process. The nanocomposite demonstrated as > 90% adsorption efficiency for ACG2 under a variety of conditions, with reusability tests showing retention of over 80% adsorption capacity after five regeneration cycles. This study focusses on the synthesis of an algae-derived biochar with magnetic properties, enhancing its efficiency in post-adsorption separation. The adsorption of Azocarmine G2 (ACG2), a hazardous azo dye, is addressed herein for the first time, establishing the novelty of this research within the domain. Furthermore, this innovative Fe_3_O_4_@BC adsorbent compound effectively resolves the issue of recyclability. The results highlight that the algal-based magnetic biochar nanocomposite is a viable and sustainable adsorbent, demonstrating exceptional dye adsorption capacity, simplified separation processes, and recyclability. Therefore, it is deemed appropriate for extensive applications in wastewater treatment processes.

**Supplementary Information:**

The online version contains supplementary material available at 10.1186/s13065-025-01474-6.

## Introduction

The expeditious industrialization and urbanization of recent decades have resulted in the emission of several synthetic organic dyes into natural aquatic systems, thereby presenting a significant environmental challenge [1, 2]. Among these dyes, azo dyes, distinguished by their azo (-N = N-) linkage, display exceptional coloration and are thus widely used [3]. Nevertheless, the structural stability of azo dyes, such as ACG2, impedes their biodegradation and renders them persistent in aquatic ecosystems, eliciting concerns regarding potential environmental contamination and adverse health implications [4]. Azocarmine G dyes have attained prominence because of their widespread application across numerous industries, including textiles, leather, and food processing. These challenges have precipitated substantial research efforts aimed at devising efficient and sustainable methodologies for the adsorption of azo dyes from aqueous milieus [5, 6]. ACG2 is one of the most perilous dyes ubiquitously employed across many essential applications, ranging from critical medical procedures to diverse industrial operations. Azocarmine G dyes are sulfonated anionic dyes with intricate chemical architecture, featuring a structure known formally as 7-Phenyl-5-(4-sulfoanilino) benzo[a]phenazin-7-ium-3-sulfonic acid, is encapsulated within its empirical formula, C_28_H_18_N_3_NaO_6_S_2_. The chromophoric azo groups undergo reductive cleavage and form highly toxic aromatic amines. Because of the electron-withdrawing groups in azo dyes, biodegradation is difficult. The azo groups undergo reductive cleavage to generate highly hazardous aromatic amines, and biodegradation is difficult because azo dyes contain electron-drawing groups. Furthermore, these reductive cleavage products are highly mutagenic and carcinogenic. Every year 175,000 tons dyes are producing among azo dyes are occupied 60% of dye generation and approximately 15% of azo dyes are released [7] into water bodies. The release of this formidable compound into the environment has the potential to cause severe environmental degradation and pose formidable health risks. Consequently, comprehensive treatment strategies should be rigorously implemented before any discharge to mitigate potentially harmful effects.

Traditional wastewater treatment processes, including membrane filtration, coagulation, chemical precipitation, flocculation, electrochemical removal, solvent extraction, biological treatment, ion exchange, etc.[8–11]. However, these methods have disadvantages i.e., incomplete removal, high energy requirement, production of secondary sludge and are inadequate for effectively addressing the adsorption of ACG2 due to its recalcitrant nature [12–14]. Adsorption is considered an efficient and low-cost technique for adsorption of dyes, organic compounds, and drugs from wastewater effluents. Furthermore, the adsorbents can be regenerated through the desorption process, because the adsorption process is reversible, which means that the adsorbates can be transferred from the adsorbent surface to the solution. However, a few challenges have been discovered, including adsorbent separation after adsorption, desorption, regeneration processes, and adsorbent stability.

Accordingly, an increasing focus has been on investigating novel materials and technologies for the efficient adsorption and elimination of these dyes. Several adsorbents are reported in the literature i.e. bentonite, kaolinite, montmorillonite, zeolites [15, 16], agricultural waste and plant-based adsorbents [17], biopolymers including chitosan, cellulose, lignin, alginate/PVA matrix [18, 19], algae, fungi, and bacterial biomass [20, 21], metal-based and inorganic adsorbents [18, 22, 23], synthetic and polymeric adsorbents [24]and biochar [25]. Linguistic cellulosic materials such as rice husk and rice straw are used as adsorbents, and other applications are organic fertiliser additive, making bricks, and production of bioethanol, biochar, and composites. Among these advanced materials, biochar, a carbon-rich porous material derived from biomass pyrolysis, has demonstrated remarkable adsorption capabilities [26]. A substantial array of organic wastes, such as agricultural wastes [17], algae (blue algae, green algae, and *Enteromorpha prolifera*), biochar-architectured chitosan [24], Poplar wood sawdust [27], oil palm frond [28], acid treated rice straw [29], rice husk [30], cassava peel [31] and Kigelia fibrous [32]can serve as raw materials for the preparation of biochar and magnetic biochar [33]. Biochar is produced via thermochemical processes such as pyrolysis, hydrothermal carbonization, and gasification [34]. The unique structural and surface properties of biochar, such as its high specific surface area, abundant functional groups, and exceptional stability, make it an attractive candidate for adsorption applications. There are several algae that were used as biosorbents, but a limited number of reports can be observed in the literature on algae biochar. The algae have abundant functional groups i.e. –COOH, –NH_2_, –OH and –SH groups on algae cell wall provide the binding sites for interaction of dyes. Furthermore, macro algae are available abundantly in the sea and sea-costs. Among Ulva fasciata it is abundantly available in sea and sea cost and it has an ulvan polymer which is responsible for the abundant functional groups. Numerous studies have documented the outstanding adsorption capacity of biochar for various organic contaminants, including dyes. For instance, in their research, Ravindiran et al. [35] demonstrated the effective adsorption of acid blue 210 &7 dye by biochar derived from sewage sludge, emphasizing the importance of biochar’s porous structure and surface functional groups in enhancing adsorption efficiency. Similarly, Zhao et al. [36] investigated the adsorption of Methylene Blue dye using bamboo-derived biochar and highlighted the role of biochar surface chemistry in the adsorption process.

Despite its potential, the practical implementation of pristine biochar in large-scale water treatment projects is significantly hindered by formidable challenges. These challenges manifest as difficulties in separation, low reusability, and limited adsorption capacity, particularly in the case of tenacious dyes like ACG2 [37]. To address these challenges, researchers have diligently developed composite materials by incorporating various functional nanomaterials into biochar matrix [38, 39]. These engineered composites aimed to leverage and integrate the robust characteristics of both biochar and incorporated nanoparticles, significantly enhancing the adsorption capabilities. Among these nanoparticles, iron oxide and zinc oxide are particularly promising candidates [40, 41]. Iron oxide nanoparticles, which are characterized by inherent magnetic properties and an extensive surface area, have consistently demonstrated exceptional potential in adsorption applications [30]. Several studies have highlighted their effectiveness in the adsorption of organic pollutants, illustrating the capability of materials based on iron oxides. However, the application of iron oxide-modified biochar for the adsorption of ACG2 has not yet been explored. This manuscript presents a detailed investigation into the preparation and characterization of an innovative biochar-iron oxide nanocomposite precisely engineered for the effective adsorption of ACG2 dye from aqueous solutions. The detailed synthesis and characterization of the composite are comprehensively discussed, with a focus on its structural and morphological properties. Additionally, this study offers a systematic evaluation of the adsorption performance of the composite, thereby elucidating the factors that influence the adsorption process.

## Materials and methods

The Ulva fasciata macroalgae was collected from the Bay of Bengal, Visakhapatnam, India. Sigma-Aldrich, Germany provides analytical–grade ACG2, NaOH, Fe_2_(SO_4_)_3_ and FeSO_4_‧7 H_2_O.

### Preparation of biochar from algae

*The Ulva fasciata algae were cleansed using distilled water to remove soil and debris, followed by air drying for 48 h.* The samples were then ground and subjected to oven drying at 343 K for 24 h. Subsequently, pyrolysis was carried out at 723 K for 2 h under a nitrogen gas flow of 10 mL/min with a heating rate of 283 K/min. The algae biochar was rinsed multiple times with ultrapure water, followed by air drying at 378 K overnight.

### Preparation of magnetic biochar (Fe_3_O_4_@BC) nanocomposites

Magnetic biochar (Fe_3_O_4_@BC) was prepared as described by Kadimpati et al. [3]. Fe^+2^/Fe^+3^ solution was prepared by mixing a ferric sulfate solution (prepared by dissolving 3.70 g of Fe_2_(SO_4_)_3_ in 260 mL of ultrapure water) and a ferrous sulfate solution (obtained by dissolving 4.00 g FeSO_4_·7H_2_O in 30 mL of ultrapure water) and then heating to 333 K while vigorously stirring. Ten grams of BC powder was gradually added to the prepared Fe^+2^/Fe^+3^ solutions and stirred for one hour. Subsequently, a solution of 5 M sodium hydroxide solution was subsequently added dropwise until reaching pH value between 11 and 12. After stirring the suspension for another hour, it was allowed to settle for a day, followed by filtration and drying at 378 K. The dried matter was then subjected to pyrolysis at 723 K for 2 h under a nitrogen gas flow of 10 mL/min with a heating rate of 283 K/min. The carbonized material was washed thoroughly with ultrapure water and oven-dried overnight at 378 K. The carbonized material was then milled, sieved, and stored for utilization (Fig. S1).

### Characterization of the prepared nanocomposites

Different tools were used for the chemical, physical, and morphological characterization of the prepared nanocomposites. X-Ray Diffraction Analysis (XRD) was carried out by (PANanlytical, X'PRT PRO). Using Cu-target with Ni-filtered radiation (λ = 1.542 A°) and the diffraction angle (2θ) was ranged between 2° and 80°. the morphology of crystals was studied using scanning electron microscopy (SEM), while energy dispersive X-ray spectrometry (EDX) was used for determination of chemical composition. The instrument used was a Jeol JSM 5300 from Japan, in which all samples were placed on stubs and gold-coated to ensure their electrical conductivity and the magnification 2500X was used. Fourier transform infrared (FTIR) spectroscopy measurements were performed using an Agilent Cary 630 instrument in the wavelength range of 400–4000 cm^−1^. The Raman spectra of the composite samples was conducted using Dispersive Raman microscope (Senterra, Brucker, US) with a laser wavelength of 532 nm at 0.2 mW.

### Adsorption of ACG2 dye

A stock solution of ACG2 was prepared by dissolving 1.0 g of ACG2 in 1 L of double-distilled water to achieve a concentration of 1000 mg/L. The stock solution was subsequently diluted to create working solutions with different ACG2 concentrations for adsorption experiments which conducted using the batch equilibrium method. In each trial, 50 mL of the ACG2 solution was mixed with a predetermined dose of the adsorbent material in a sealed container. The mixtures were then agitated in a mechanical shaker to facilitate adsorption. Following agitation, the solutions were centrifuged, and the supernatant was analyzed using a visible–UV spectrophotometer (UV5600, Shanghai Metash Instruments Co., Ltd) to determine the residual ACG concentration. Absorbance measurements were taken at a wavelength of 516 nm, which corresponds to the maximum absorption peak of ACG2. The adsorption capacity (q_t_) of the adsorbent at time t was calculated using the following equation:

1 $$\text{Adsorption capacity}, {q}_{t}=\frac{{(C}_{0}-{C}_{t})v}{m}$$

where C_0_, C_t_, are ACG2 concentrations at initial, and at equilibrium time (mg/L); qt is adsorption capacity at time t (mg/g); m is the adsorbent mass (g); v is the solution volume (L).

The adsorption percentage of ACG2 dye from aqueous solution is calculated using the following equation.

2 $$\text{ACG}2\text{ \% removal}=\frac{{C}_{0}-{C}_{t}}{{C}_{0}}\times 100$$

The pH effect on the adsorption of ACG2 using nanocomposite as an adsorbent was investigated. Specifically, 50 mg of the magnetic biochar was added to 50 mL of a 100 mg/L ACG2 dye solution, at initial pH values of 1, 1.5, 2, and 2.5. pH adjustment was done using 0.1 M HCl and NaOH solutions. The mixture was subsequently stirred at 160 rpm over several time periods (15, 30, 45, 60, 90, 120, 180, and 300 min) at ambient temperature, followed by the collection of samples for analyzing the color of ACG2 dye. An isotherm study was also conducted using different concentrations of ACG2 dye solutions (25, 50, 75, 100, and 150 mg/L). For this purpose, 50 mL of ACG2 solutions with various initial concentrations were agitated with the adsorbent at 160 rpm for 180 min at 298 K. The effects of BC dose and contact time on ACG2 dye adsorption were examined by agitating 50 mL of ACG2 dye solutions with initial concentrations of 75, 100, 150, and 200 mg/L (for both BC and Fe_3_O_4_@BC) with different adsorbent doses (1.5, 2, 2.5, and 3 g/L) for various time intervals (0, 15, 30, 45, 60, 90, 120, 180, and 300 min) at 298 K.

### Kinetic modeling, adsorption isotherms, and thermodynamics

The adsorption of ACG2 onto Fe_3_O_4_@BC was analyzed using pseudo-first-order (PFO), pseudo-second-order (PSO), and intraparticle diffusion (IPD) kinetic models. The linear form of the PFO, PSO and IPD models is as follows (Eq. 3–5):

3 $${\text{Pseudo first order}}:\,\ln \left( {q_{e} - q_{t} } \right) = \ln q_{e} - k_{PFO}^{t}$$

4 $${\text{Pseudo Second order}}\frac{t}{{q_{t} }} = \frac{1}{{k_{PSO} \times q_{e}^{2} }} + \frac{1}{{q_{e} }}$$

5 $${\text{Intraparticle diffusion}}:q_{t} = k_{ID} \times t^{\frac{1}{2}} + C$$

In this context, qt and qe represent the quantities of ACG2 adsorbed per unit weight on the Fe_3_O_4_@BC nanocomposite at a given time ‘t’ and at equilibrium, respectively. The kinetic constants kPFO, kPSO, and kID correspond to the PFO, PSO, and IPD models, respectively, and are determined by evaluating the slopes of the plots of In(qe-qt) versus t, t/qt versus t, and qt versus t^1/2. The adsorption capacities of the nanocomposite for ACG2 were assessed employing the Freundlich and Langmuir isotherm models. The Freundlich model presupposes non-ideal adsorption of ACG2 on heterogeneous surfaces, whereas the Langmuir isotherm presumes monolayer adsorption on the adsorbent surface. The linearized equations of these models are presented as follows (Eq. 6–7):

6 $$Freundlich \, isotherm:\,\log q_{e} = \log K_{F} + \frac{1}{n}\log C_{e}$$

where, Ce and qe represent the equilibrium concentration (mg L-1) and equilibrium adsorption capacity (mg g^−1^) of ACG2, respectively, while KF[(mgg^−1^)(Lmg^−1^)1/n] serves as a constant indicative of the adsorption capacity associated with the adsorption process. The Freundlich model posits that the adsorption of ACG2 occurs on heterogeneous surfaces, reflecting a non-ideal sorption mechanism. The Freundlich constant 'n', indicative of adsorption intensity, is deemed more favorable when values fall within the range of 1 to 10. By examining the slope of the plot of log (qe) versus log (Ce), it is possible to determine the Freundlich parameter values, expressed as 1/n and KF.

7 $$Langmuir \, isotherm:\,\frac{{C_{e} }}{{q_{e} }} = \frac{1}{{q_{m} \times K_{L} }} + \frac{{C_{e} }}{{q_{m} }}$$

where, qm represents the maximum adsorption capacity of the Fe_3_O_4_@BC nanocomposite, while KL denotes the Langmuir equilibrium constant (L mg⁻^1^). The Langmuir isotherm presupposes the monolayer adsorption of ACG2 on the adsorbent’s surface. The parameters qm and KL can be derived from the slope of the plot of 1/qe against 1/Ce. Furthermore, the thermodynamic parameters of adsorption were evaluated over varying temperatures (298–328 K), encompassing calculations of ΔG (Gibbs free energy, J mol⁻^1^), ΔH (enthalpy, kJ mol⁻^1^), and ΔS (entropy, J mol⁻^1^ K⁻^1^) associated with ACG2 adsorption on the adsorbents, as referenced in. [26]:

8 $$\Delta G^{o} = - RT{\text{ In }}K_{C}$$

9 $$K_{C} = \frac{{C_{S} }}{{C_{e} }}$$

10 $$\Delta G^{o} = \Delta H^{o} - T\Delta S^{o}$$

11 $$Log\left( {\frac{{q_{e} }}{{C_{e} }}} \right) = \frac{{\Delta S^{o} }}{2.303R} - \frac{{\Delta H^{o} }}{2.303RT}$$

where R represents the universal gas constant, T denotes the temperature in Kelvin, CS signifies the concentration of ACG2 on the adsorbent measured in milligrams per gram, and Ce is the equilibrium concentration of ACG2 in milligrams per liter. The values of ΔG°, ΔH° and ΔS° are calculated from slope of Log (qe/Ce) vs. 1/T.

### Regeneration and reusability of Fe_3_O_4_@BC

The regeneration and reusability of Fe_3_O_4_@BC for the adsorption of ACG2 from water were evaluated. After the adsorption process, the nanocomposites were washed with a desorption medium consisting of 100 mL of 99% ethanol [3] under continuous agitation for a duration of 4 h at a temperature of 298 K. Subsequently, the biochar adsorbents were separated, dried, and reutilized in successive cycles. These adsorption-regeneration cycles were conducted five times utilizing the same regenerated nanocomposite, and the quantity of ACG2 desorbed was determined using Eq. (12).

12 $$q_{des} = C_{des} \frac{V}{W}$$

where V is the solution volume, q_des_ is the desorption quantity, C_des_ is the desorption concentration, and W is the weight of the nanocomposite.

The desorption ratio was also estimated using Eq. (13).

13 $${\text{Desorption ratio}} = \frac{{\text{Amount of ACG2 sorbed}}}{{\text{Amount of ACG2 desorbed}}} \times 100$$

## Results and discussion

### Characterization of the synthesized BC and Fe_3_O_4_@BC

#### XRD

**Fig. 1:**
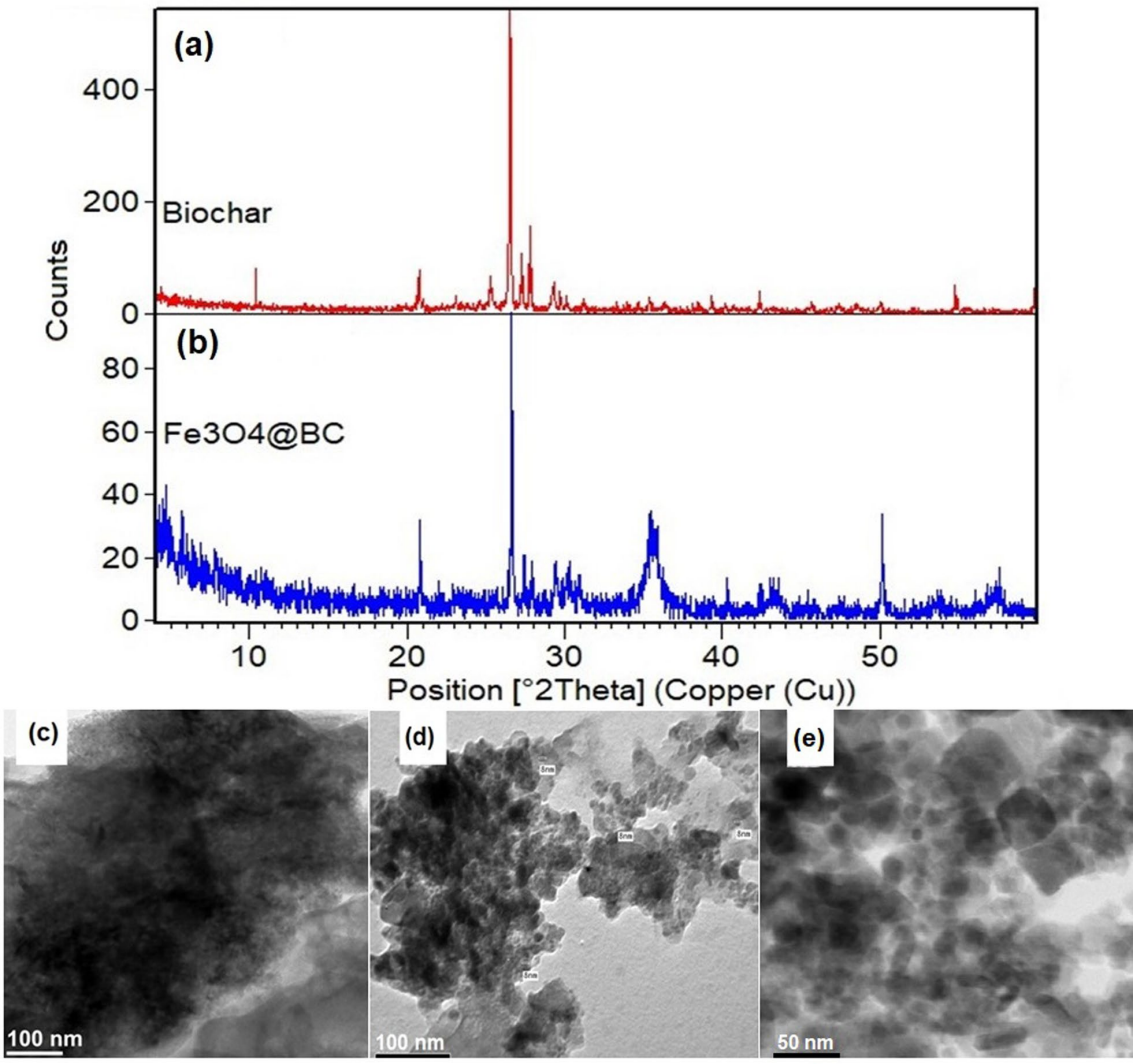
XRD analysis of BC (**a**), Fe_3_O_4_@BC (**b**), structural analysis of the TEM images of BC (**c**), Fe_3_O_4_@BC (**d**) and Fe_3_O_4_@BC-ACG2 (**e**)

The XRD patterns of BC and Fe_3_O_4_@BC are presented in Fig. 1a, b. The BC (algal biochar) pattern shows characteristic graphite-like crystalline carbon peaks at 2θ = 25.9° and 43.9°. Notably, CaO peaks appear at 2θ = 31.3°, 36.3°, and 47.3°, while CaCO_3_ peaks were evident at 2θ = 23.0°, 29.3°, 35.9°, 43.2°, 47.5°, 48.5°, and 59.99°. These results indicate the presence of CaO and CaCO_3_ in the BC along with low-intensity graphite-like peaks (Fig. 1a). This proposes BC was carbonated with CO_2_ to form CaCO_3_. Moisture and CO_2_ readily react with CaO to produce Ca(OH)_2_ and CaCO_3_, respectively. The CO_2_ reacted with CaO and Ca(OH)_2_ to form thermodynamically stable CaCO_3_, as described by Eqs. (4) and (5). CO_2_ acts as an activation agent, generating numerous small porous structures in biochar, consistent with the results of Shi et al. [42].

14 $$\text{CaO}(\text{s}) + {\text{CO}}_{2} \to \text{ Ca}{\text{CO}}_{3}(\text{s})$$

15 $${\text{Ca}(\text{OH})}_{2}(\text{s}) + {\text{CO}}_{2} \to {\text{CaCO}}_{3}(\text{s}) + {\text{H}}_{2}\text{O}$$

The Fe_3_O_4_@BC magnetic nanocomposite XRD pattern shows (Fig. 1b) peaks at 21.1°, 35°, 41.3°, and 50.3°, which are assigned to the crystalline structure of Fe_3_O_4_@BC (JCPDS 19–0629). The peak at 2θ = 35.31° was attributed to the incorporation of Fe_3_O_4_@BC, while peaks at 2θ = 30° and 43° were generated by the interaction of Fe_3_O_4_@BC and BC. Comparison with JCPDS card no. 36–1451 suggests a polycrystalline hexagonal phase (wurtzite type), which is consistent with previous research [43, 44].

#### TEM study of Fe_3_O_4_@BC nano-composite

TEM images of BC and Fe_3_O_4_@BC are presented in Fig. 1c, d. The BC surface is rough (Fig. 1c) and the Fe_3_O_4_@BC nanocomposite, a magnetic component was embedded in the carbon layer (Fig. 1d). The material has an irregular shape (some of the particles appeared as spherical, oval and tetragonal shapes), with the magnetic component distributed unevenly covered by the carbon layer. The Fe_3_O_4_@BC core had a mean diameter of 8 ± 2.8 nm. The iron oxide particles were embedded in BC matrix was, indicating good mechanical bonding. The dispersion of iron oxide particles was observed in BC was consistent with that of previous studies [45]. Further, after adsorption the TEM image showing that the particles are darkened, and morphology is also changed due to the ACG2 covered on particles (Fig. 1e). Selective area electron diffraction (SAD) analysis identified these two main components, with the iron oxide nanoparticles exhibiting significant crystal lattice planes and the surrounding biochar being amorphous (Figure S2). The SAD pattern shows clear, sharp diffraction rings, indicating the polycrystalline nature of the iron oxide. The XRD pattern (Fig. 1) confirms that the iron oxide particles were mainly magnetite, with minimal additional phases.

#### SEM and elemental analysis

**Fig. 2:**
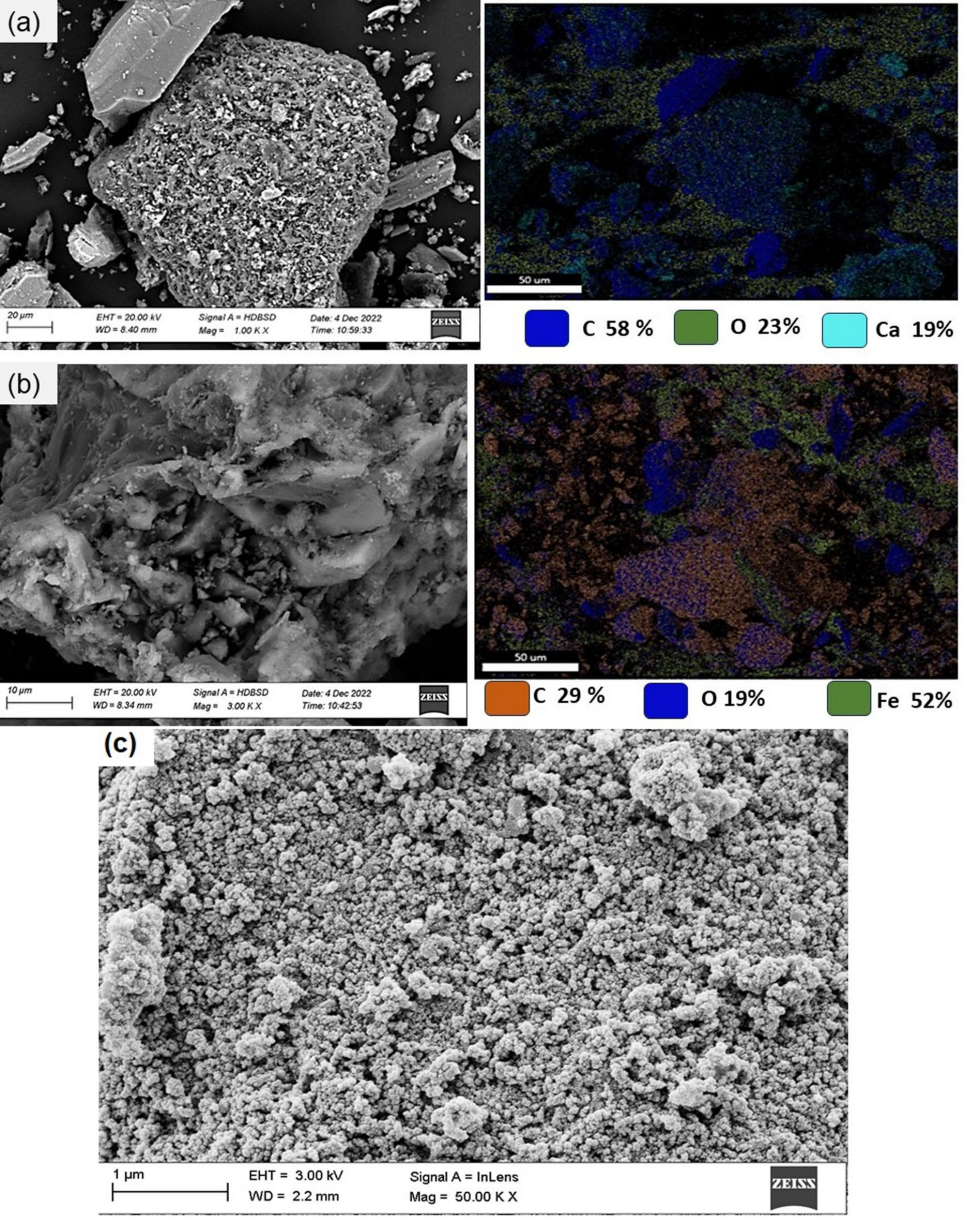
SEM micrographs and EDX-mapping for elements of BC (**a**), Fe_3_O_4_@BC (**b**) and Fe_3_O_4_@BC-ACG2 (**c**)

SEM micrographs of BC, Fe_3_O_4_@BC and Fe_3_O_4_@BC-ACG2 are shown in Fig. 2. EDX and map analyses were used to observe elemental distribution on the surface morphology and changes in biochar produced from algae BC and, Fe_3_O_4_@BC magnetic nanocomposite, as indicated in the EDX analysis in Figure S3. As shown in Fig. 2a, SEM micrographs show that BC produced from marine algae biomass has a rough and porous surface with different pore diameters. It is clearly demonstrated that BC had an uneven shape with a wrinkled surface. EDX-mapping showed that elements C (52 W%), O (23 W%), and Ca (19 W%) were evenly distributed on the surface of BC. It should also be noted that the XRD analysis confirmed the presence of CaO and CaCO_3_ particles in the BC composite structure., there are many irregular pores in the biochar, which are facilitated to be incorporated with Fe_3_O_4_ magnetic nanoparticles, as shown in Fig. 2b. Further, after adsorption of ACG2 the Fe@BC nanocomposite rough and porous surface is covered with a dark matter, probably due to ACG2 adsorption on to the porous-like structure appeared to be filled can be seen in Fig. 2c. EDX analysis showed that carbon and Iron are the most active elements in the Fe@BC nanocomposite and observed the Fe and C are 49 and 29% respectively. The EDX results of BC, Fe_3_O_4_@BC and Fe_3_O_4_@BC –ACG2 were depicted in Figure S3. After adsorption of ACG2 the EDX results showed that the increase in weight percent of oxygen and carbon elements whereas Fe is decreased (Figure S3). This may be due to covering of carbon and oxygen species on the iron (Figure S3c). These results are indicated that the adsorption of ACG2 occurred on Fe_3_O_4_@BC nanocomposite.

#### Study on functional groups using FTIR

**Fig. 3:**
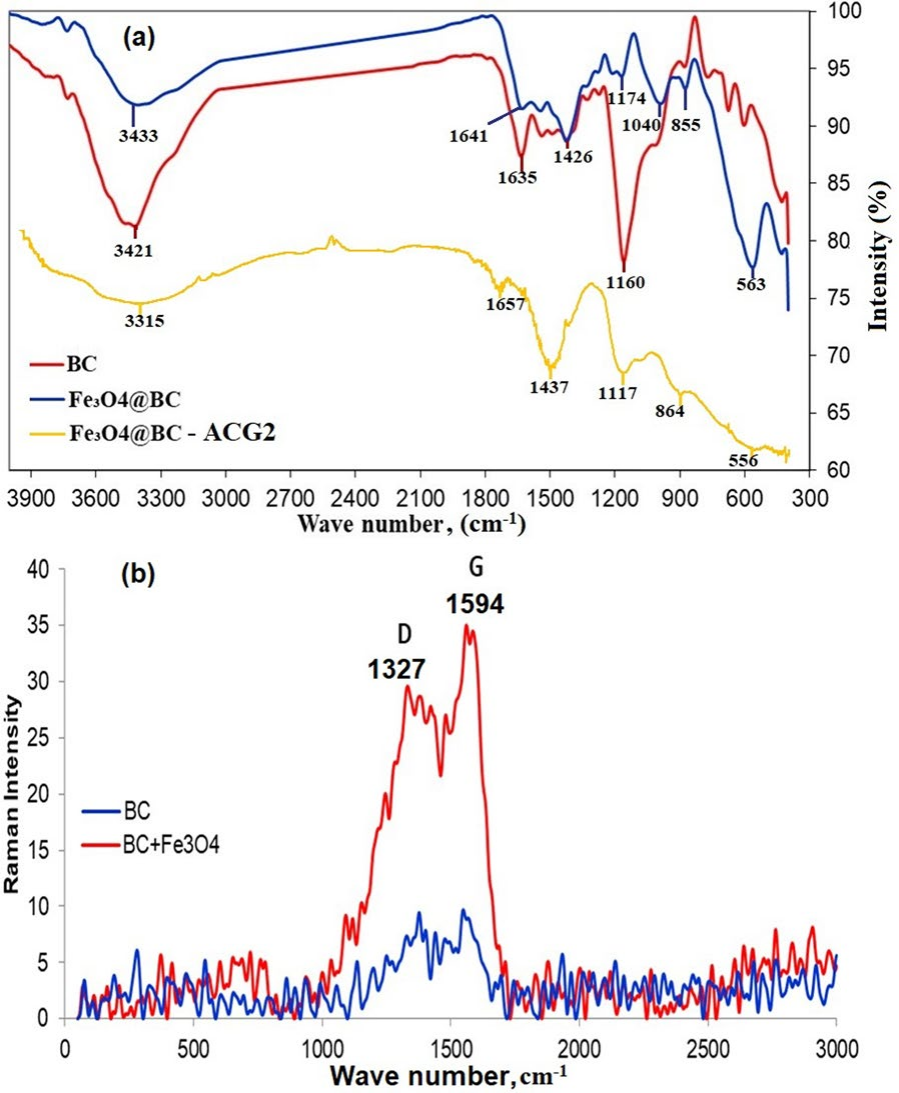
**a** FTIR analysis of BC, Fe_3_O_4_@BC, and Fe_3_O_4_@BC–ACG2 and (**b**) Raman analysis of BC and Fe_3_O_4_@BC

The FTIR spectra of BC and Fe_3_O_4_@BC are presented in Fig. 3a. The BC spectrum shows peaks at 3421, 1635, 1426, and 1160 cm^−1^, attributed to the vibration of –OH, C = O, carboxyl O = C–O, and alkoxy C–O groups, respectively. The broad band between 3200 and 3600 cm^−1^ is likely due to the vibration of the hydroxyl group (^−^OH) of cellulose-derived products. The FTIR results revealed an abundance of oxygen-containing functional groups (C = O, O–H, C-O), which can act as electron donors [46]. The hydroxyl and carboxyl groups on the biochar surface play crucial roles in pollutant adsorption [47]. The Fe_3_O_4_@BC composite has functional groups (-OH, C = O, Fe–O) that are effective for dye adsorption via chemical bonds [48]. After development of Fe_3_O_4_@BC composite the hydroxyl functional group at 3421 cm^−1^ (sharp peak) on biochar is shifted to 3433 cm^−1^ and formed broad peak (Mosaffa et al. 2024). The carboxylic group peak is also shifted 1635 to 1641 cm^−1^ (Mosaffa et al. 2025) and C–O groups at 1160 shifted to 1174 cm^−1^ due to the indicated that the involvement in the Fe_3_O_4_@BC composite formation (Fig. 3a). Upon the incorporation of Fe_3_O_4_ into BC, a new adsorption band appeared at 563 cm^−1^, attributed to the Fe–O stretching vibration, confirming the successful preparation of the magnetic biochar composite [3].

After adsorption of ACG2 on Fe_3_O_4_ @BC, the peak of 3433 cm^−1^ is shifted to 3315 cm^−1^ and formed broad peak due to the involvement of hydroxyl functional group in the adsorption process. The sharp peak at 1641 cm^−1^ due to the presence of C = C shifted to 1657 cm^−1^ attributed to the participation in the adsorption process. The bands at 1437 and 864 cm^−1^ correspond to the bending vibration of C–H and the out-of-plane deformation of aromatic CH, respectively after adsorption of ACG2. Similarly, a new peak was observed at around 1117 cm^−1^ due to C–N on due to azo group of ACG2 on Fe_3_O_4_ @BC after adsorption process (Fig. 3a). Algal biochar and dye interactions can be explained by the following: (1) cationic dye and negatively charged carboxylate group interaction, (2) coordinate bond between the lone pair electrons of the OH or NH_2_ group and the dye, (3) Van der Waals forces between the nonpolar groups of both algal cell and dye, and (4) ion–dipole bond between the dye molecule and the negative dipole end of the carbonyl group. Adsorption can occur via chemical (chemisorption) or physical mechanisms. Chemisorption involves the formation of chemical bonds between the adsorbate molecule (dye) and the adsorbent surface (biochar). Physical adsorption relies on physical forces like van der Waals forces. A larger surface area can accommodate more adsorbate molecules via physical interactions. The porous structure also enables adsorbate diffusion.

#### Raman spectra

The Raman spectra of the BC, and Fe_3_O_4_@BC, composites are depicted in Fig. 3b. Prominent peaks appear in the ranges of 1327–1380 cm^−1^ and 1550–1594 cm^−1^, assigned to the D and G energy bands, respectively [49]. The D band is associated with sp^3^ carbon (C) atom vibrations arising from local disorders and defects, while the G band relates to the tensile vibrations of sp2 atoms in a graphite hexagonal lattice [50]. The ratio of the D and G band intensities (ID/IG) can describe graphite material structural irregularities [51]. The ID/IG ratios for BC and Fe_3_O_4_@BC were 0.7095 and 1.0372, respectively. A high ID/IG ratio indicates more defects and increased functionalities. The ID/IG ratio for BC/Fe_3_O_4_ was higher than that for BC, indicating that the BC structure was modified by Fe_3_O_4_ nanoparticles. This increase in the ID/IG ratio also suggests a reduction in BC content after the formation of the Fe_3_O_4_@BC magnetic composite [52].

#### Determination of texture properties via BET

The textural properties (specific surface area, total pore volume, pore size distribution) were investigated using N_2_ adsorption–desorption isotherms. The surface area is a critical indicator of the adsorbent capacity. Results are depicted in Table 1 and supplementary Figure S4(a,b). The BC has a specific surface area of 3.80 m^2^/g and displays a type-II isotherm, indicating a nonporous structure with abundant micropores and mesopores [53, 54]. This suggests a hierarchical porous structure consisting of micropores and mesopores in the Fe-BCs, as confirmed by the pore size distribution. This structure can minimize the mass transfer resistance and facilitate the transport of reactants to the catalysts [55]. The specific surface areas and total pore volumes of BC and Fe_3_O_4_@BC were 3.804 m^2^/g and 0.013 cm^3^/g and 27.411 m2/g and 0.113 cm^3^/g, respectively (Table 1). Algal biomass-derived biochar is known to have a lower surface area. Surface area is the primary physical factor affecting biochar dye sorption. As shown in Table 1, the BC BET area increased from 3.804 m^2^/g to 51.92 m^2^/g after magnetization with iron oxide. The large Fe_3_O_4_@BC surface area likely provides numerous active adsorption sites. Similar results were observed for the pore volume. Compared with BC (0.015 cm^3^/g), the Fe_3_O_4_@BC pore volume (0.153 cm^3^/g) increased by roughly tenfold, suggesting that Fe introduction potentially enhances Fe_3_O_4_@BC porosity. The average pore sizes of BC and Fe_3_O_4_@BC were 8.526 and 5.804 nm, respectively (Table 1). The BC and Fe_3_O_4_@BC BET surface areas are lower than those of most commercial adsorbents, such as activated carbons (1896 m^2^/g) [56]. The Fe_3_O_4_@BC pore diameter range of 3.12–3.6 nm indicates mesoporosity.

**Table 1:** Textural properties of BC and Fe_3_O_4_@BC

Samples	Specific surface area (m^2^/g)	Total pore volume (cm^3^/g)	Micropore volume (cm^3^/g)	Mesopore volume (cm^3^/g)	Pore volume (cm^3^/g)	Pore diameter (nm)
BC	3.804	0.013	0	0.013	0.015	3.618
Fe_3_O_4_@BC	51.92	0.113	0	0.113	0.153	3.609

### Adsorption of ACG2 on BC and Fe_3_O_4_@BC

#### Effect of contact time on ACG2

The effect of contact time on ACG2 adsorption at concentrations of 100 and 50 mg/L using BC and Fe_3_O_4_@BC adsorbents with a BC concentration of 2 g/L was examined over a duration of 6 h. Equilibrium was achieved after 220 min, as demonstrated in Supplementary Figure S5a, b. Consequently, an equilibrium time of 4 h was established for all subsequent experiments. Additionally, adsorption studies were conducted over a pH range of 1–10.

#### The effect of pH on adsorption of ACG2

**Fig. 4:**
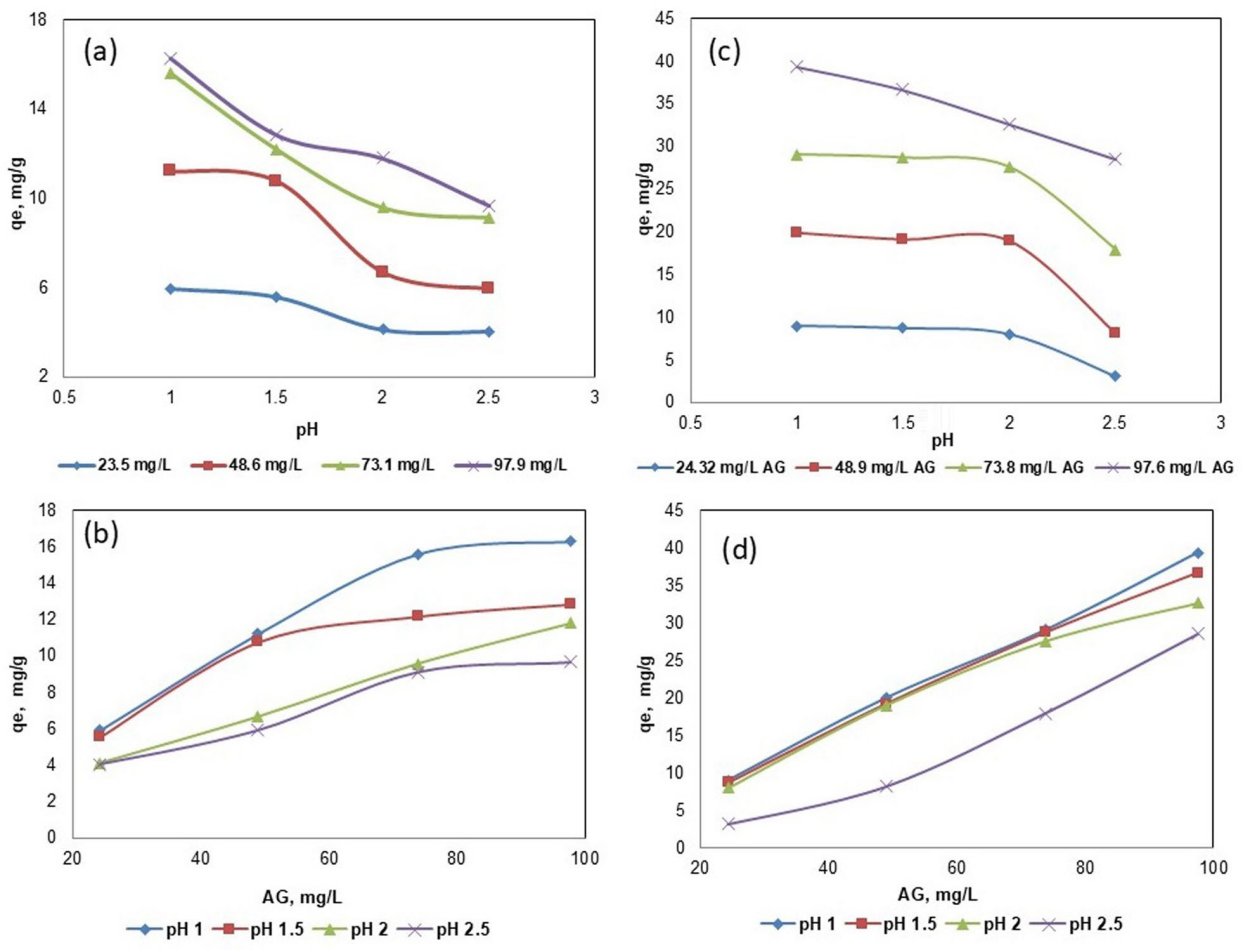
Influence of initial pH on adsorption efficiency of (**a**, **b**) BC and (**c**, **d**) Fe_3_O_4_@BC at different ACG2 concentrations

The initial investigations revealed that ACG2 adsorption was most effective under acidic conditions, with an optimal pH range of 1–2.5, as depicted in Fig. 4. The maximum adsorption capacities (q_e_) for various ACG2 concentrations in BC were reported as 5.92, 11.2, 15.584, and 16.28 mg/g for initial ACG2 concentrations of 25, 50, 75, and 100 mg/L, respectively, at pH 1 (Fig. 4b). Figure 4a further indicates that the highest q_e_ (mg/g) at pH 1 occurred at an initial ACG2 concentration of 100 mg/L.

The point zero charge (pH PZC) is noted at pH 2.1 on Fe_3_O_4_@BC indicated that the below 2.1 the favorable pH for the adsorption of ACG2 (Supplementary Figure S6). ACG2 is anionic dye and can be exists negative charge and at low pH the positive charges are abundantly available on the Fe_3_O_4_@BC. The integration of marine algal-based BC with magnetic nano-iron oxide showed an increase in ACG2 adsorption capacity by the double at lower pH, indicating the high activity Fe_3_O_4_@BC at high acid medium. At elevated pH levels, the neutralization of the positive charges on Fe_3_O_4_@BC occurs, accompanied by an increase in surface negative charges. This results in electrostatic repulsion between anionic dye molecules and the negatively charged Fe_3_O_4_@BC surfaces, leading to a reduction in the adsorption capacity of the Fe_3_O_4_@BC nanocomposite. Consequently, a favorable pH of 1 is indicated, as similar observations have been reported in the case of Congo red dye adsorption on cationic-amino modified banana leaves [57].

At low pH values, the surface of the magnetic biochar tends to be positively charged due to the protonation of functional groups. This condition enhances the electrostatic attraction between the positively charged surface and the anionic dye ACG2, leading to increased adsorption rates [58]. The maximum adsorption efficiency was observed at pH levels below 2, where nearly 86.8% adsorption was achieved within the first 75 min of contact. Similar findings were reported by Li et al.[59], who noted that acidic conditions significantly improved the adsorption capacity of various adsorbents for anionic dyes due to favorable electrostatic interactions.

#### Influence of adsorbent dosage

**Fig. 5:**
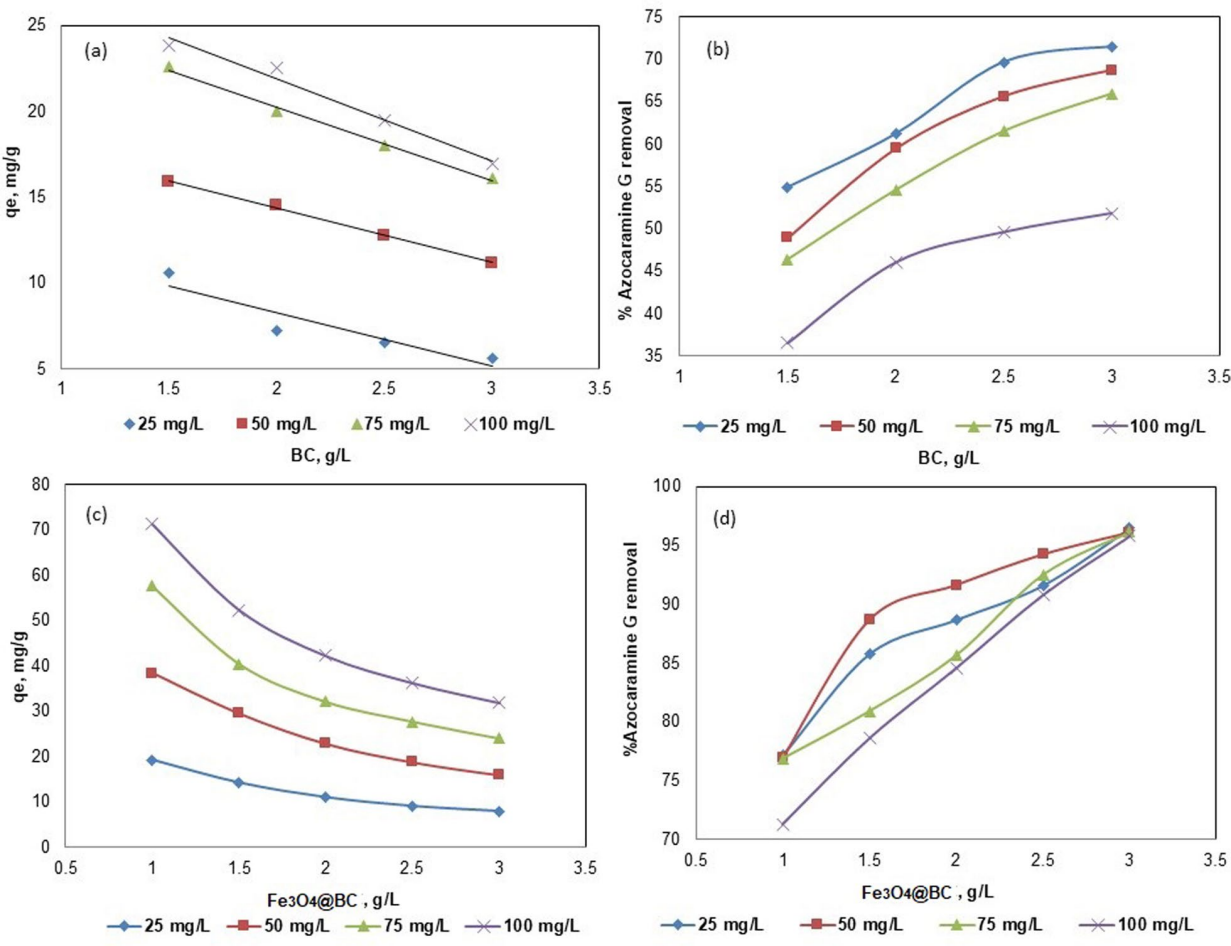
Influence of initial ACG2 concentrations on adsorption efficiency of (**a**, **b**) BC and (**c**, **d**) Fe_3_O_4_@BC at 2 g/L dosage

The adsorption of ACG2 onto BC (Fig. 5a, b) and Fe_3_O_4_@BC (Fig. 5c, d) was evaluated at various dosages (1, 1.5, 2, 2.5, and 3 g/L) at an equilibrium time of 4 h, temperature of 298 K, and pH of 1. The results presented in Fig. 5 indicate a clear trend in the relationship between the adsorbent dosage and the adsorption efficiency of the dye from aqueous solutions. In the case of modified BC with magnetic iron oxide (Fe_3_O_4_@BC), the adsorption capacities increased to 8.97, 19.94, 29.025, and 39.35 mg/g at 25, 50, 75, and 100 mg/L of ACG2 (Fig. 5c, d). The results revealed that at low dosages (1 and 1.5 g/L), the adsorption efficiency of different was in the range of only 35–55%, for BC and 70–78% for Fe_3_O_4_@BC, indicating that there were insufficient active sites on the magnetic biochar to effectively adsorb ACG2. However, these small doses showed high q_e_ values, which reached 23.8 mg/g for BC and 71.3 mg/g for Fe_3_O_4_@BCat at an initial dye concentration of 100 mg/L. The maximum adsorption efficiency was achieved when 2.5 and 3 g/L of Fe_3_O_4_@BC were used, with adsorption efficiencies ranging from 91 to 95% at different ACG2 concentrations (Fig. 5d). However, the absorption capacities at these high doses showed lower values (27.1–36.9 mg/g) at different dye concentrations (Fig. 5c). When evaluating the adsorption of ACG2 onto Fe_3_O_4_@BC, it is essential to understand the dynamics at play when high concentrations of the adsorbent are used. The observed low adsorption capacity coupled with high adsorption efficiency at elevated Fe_3_O_4_@BC dosages can be attributed to several interrelated factors. At higher doses of magnetic Fe_3_O_4_@BC, the total number of available adsorption sites increased, which initially enhanced the adsorption efficiency. However, as the concentration of biochar increases beyond an optimal point, many of these sites become saturated with dye molecules [60]. This saturation leads to a plateau in the adsorption capacity, where the additional biochar does not correspondingly increase the amount of dye adsorbed. For instance, studies have shown that once a certain adsorbent threshold is reached, the efficiency may stabilize or even decline due to saturation effects [28]. The diffusion rate of dye molecules toward the adsorbent surface may slow down as more particles are present in solution, creating a barrier for effective adsorption. This phenomenon can lead to a situation in which despite high adsorption efficiencies being recorded (indicating many molecules have been removed from solution), the actual amount adsorbed per gram of biochar is lower than expected due to these kinetic limitations [61].

#### Impact of temperature on ACG2 adsorption

The impact of temperature on the adsorption of ACG2 using BC and Fe_3_O_4_@BCat a concentration of 2.5 g/L and pH 1 was assessed at various initial dye concentrations, as shown in Supplementary Figure S7. The results indicated that increasing the temperature from 298 to 328 K had a negligible effect on the adsorption capacity of BC and Fe_3_O_4_@BC. At temperatures of 298, 308, 318, and 328 K and ACG2 concentrations of 25, 50, 75, and 100 mg/L, qe values ranges were 8.9–9.2 mg/g, 17.3–18.2 mg/g, 25.5–27.3 mg/g and 33.8–36.8 mg/g, respectively. The corresponding adsorption efficiencies were 91–95%, 90–95%, 88–94.5% and 86–93%. This low effect of temperature on the adsorption capacity can be attributed to several interrelated factors, including the nature of adsorption interactions (physisorption vs. chemisorption), compensatory mechanisms at play during kinetic energy increases, limited influence on microporous structures, and specific experimental conditions or models used for analysis. Physisorption, which is characterized by weak van der Waals forces, may show less sensitivity to temperature changes compared to chemisorption, which involves `stronger chemical bonds [62]. In cases in which physisorption dominates, the increase in kinetic energy at higher temperatures does not significantly enhance desorption rates, leading to a stable adsorption capacity across a range of temperatures [15]. In the adsorption process using porous materials like magnetic biochar, micropore filling is essential. Temperature minimally affects the microporous volume and structure, keeping the adsorption capacity stable because of the unchanged pore structures [63]. Functional groups on magnetic biochar, such as -OH and -COOH, help offset temperature effects by sustaining dye molecule affinity, stabilizing adsorption performance [64].

### Adsorption kinetics, isotherms and thermodynamics

**Fig. 6:**
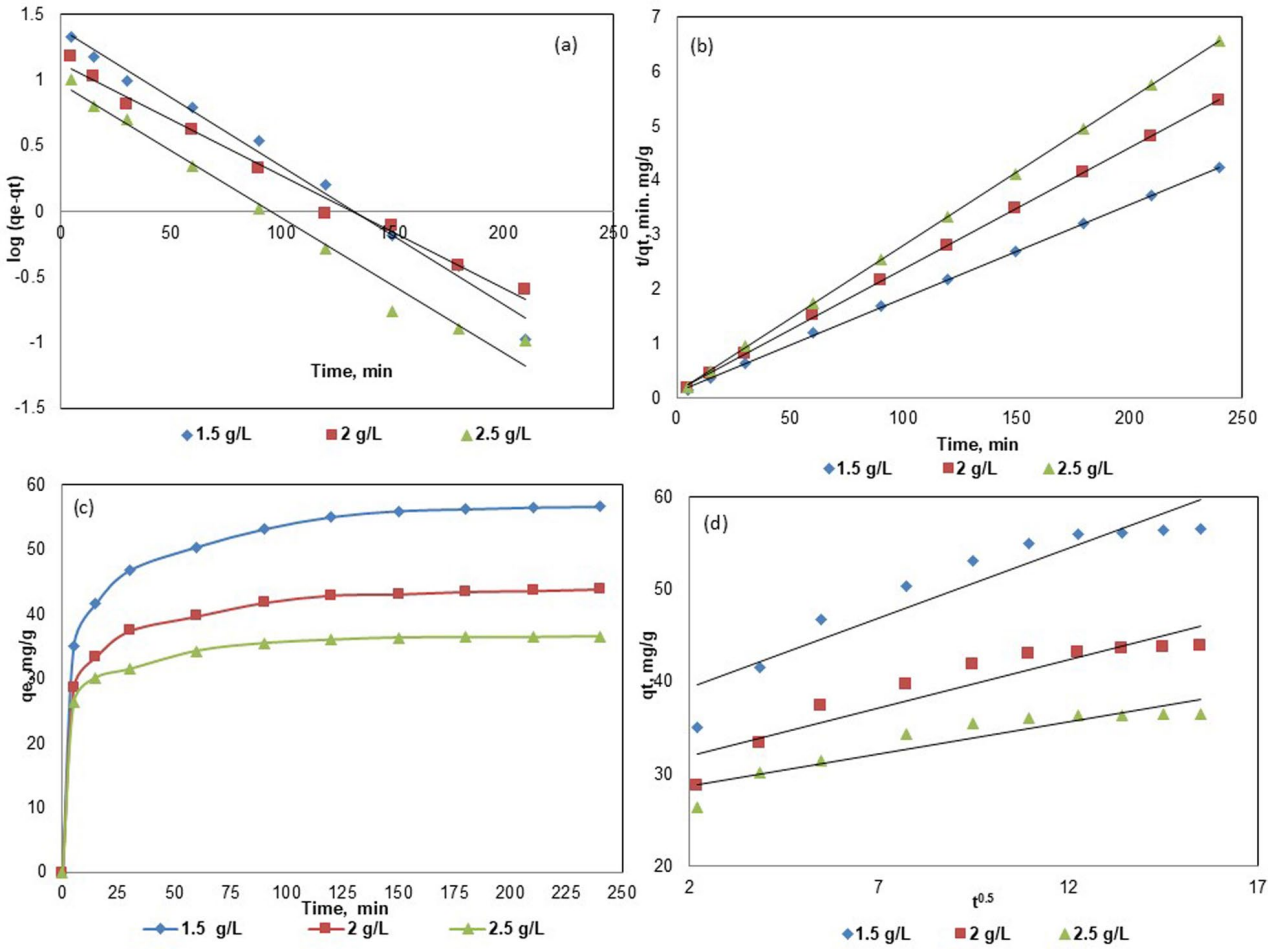
Results of kinetic study for adsorption of ACG2 on Fe_3_O_4_@BC: pseudo first-order kinetics for (**a**); pseudo second-order kinetics for (**b**); experimental q_e_ (**c**) and intra particle diffusion kinetics (**d**)

The adsorption kinetics were modeled using pseudo-first-order (PFO) and pseudo-second-order (PSO), as shown in Fig. 6. The kinetic parameters of PFO for various amounts of Fe_3_O_4_@BC were assessed based on the respective graphs (Fig. 6a). The calculated q_e_ values were lower than the experimental qe values, indicating that the PFO model does not accurately predict the adsorption kinetics of ACG2 on Fe_3_O_4_@BC. The PSO model provided the best fit for AGC2 adsorption, with an R^2^ value of 0.999 (Fig. 6b). The calculated qe values were very close to the experimental values, and the kinetic experimental data were effectively interpreted by the PSO (Table 2); and experimental q_e_ (Fig. 6c) are depicted and this conclusion is consistent with previous studies [65, 66] on modified biochar and seaweed biochar for dye sequestration. The plots in Fig. 6d show that the adsorption of ACG2 on Fe_3_O_4_@BC versus t^1/2^ did not form linear plots and did not pass through the origin. This conclusion confirmed that the rate-controlling step did not involve intra-particle diffusion; this observation aligns with those reported in the literature [65, 67].

**Table 2:** Kinetic parameters of the pseudo-first, pseudo-second order kinetic models and intra-particle diffusion at pH = 1, T = 298 K, and an initial ACG2 concentration of 100 mg/L for BC and Fe_3_O_4_@BC, respectively

Kinetic model	Biochar	q_e_ (mg g^−1^)	k_1_(min^−1^)	Experimental q_e_ (mg g^−1^)	r^2^
Pseudo-first-order	BC	13.4	0.018	12.4	0.988
	Fe_3_O_4_@BC	45.4	0.004	36.5	0.979
Pseudo-second-order	BC	24.5	0.023	18.5	0.988
	Fe_3_O_4_@BC	58.8	0.0026	51.2	0.999
Intra-particle diffusion		**k**_**P**_** (**mg h^0.5^ g^−1^)	**C**		**r**^**2**^
	BC	0.7	27.26	––	0.856
	Fe_3_O_4_@BC	1.516	36.24	––	0.885

*K*_*p*_ intraparticle diffusion rate constant, *C* constant gained from the intercept, *r*^2^ correlation coefficient

**Fig. 7:**
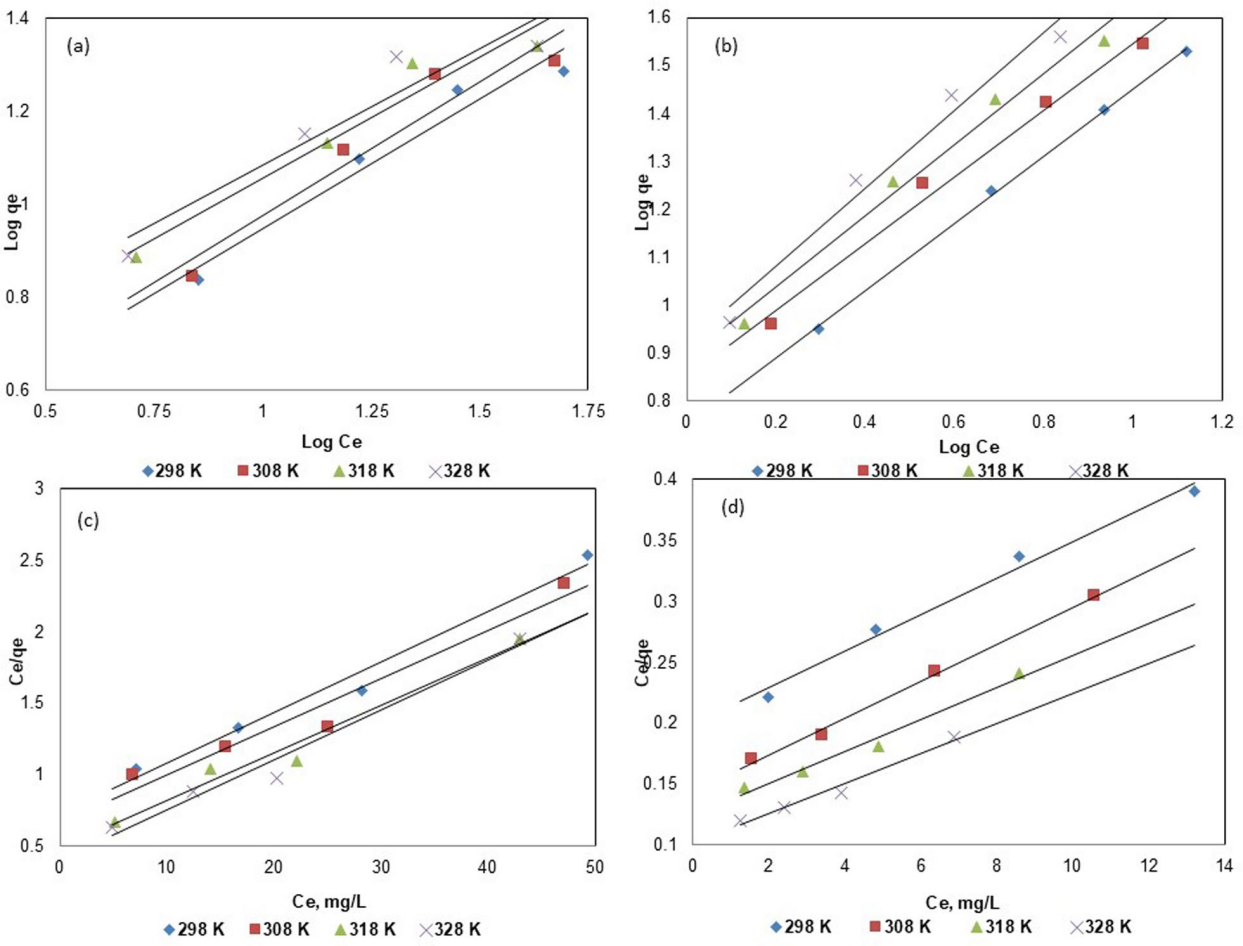
Freundlich isotherm for BC (**a**) and Fe_3_O_4_@BC (**b**), Langmuir isotherms for BC (**c**) and Fe_3_O_4_@BC (**d**) at different temperatures

The adsorption data of ACG2 by BC and Fe_3_O_4_@BC were simulated and fitted to both Langmuir and Freundlich isotherm models (Fig. 7). The corresponding isotherm parameters and their correlation coefficients (R^2^) are presented in Table 3. The Freundlich constant K_f_ decreased with increasing temperature for both BC (Fig. 7a) and Fe_3_O_4_@BC (Fig. 7b), suggesting enhanced adsorption capacity at room temperature. Furthermore, the Freundlich exponent n exceeds 1, signifying a favorable adsorption process. The superior fit of the Langmuir isotherm suggests that the adsorption behavior is predominantly monolayer, involving both chemical and physical adsorption mechanisms. These findings are consistent with previous reports on azo-dye adsorption by modified biochar [65].

**Table 3:** Langmuir and Freundlich models of ACG2 adsorption isotherms on BC and Fe_3_O_4_@BC at different temperatures

Adsorbent		Langmuir model				Freundlich model		
	*T* (K)	*q*_*m*_ (mg/g)	*b* (L/mg)	R_L_	R^2^	*K*_*f*_ [(mgg^−1^) (Lmg^−1^)^1/n^]	*n*_*f*_	R^2^
BC	298	28.57	0.0483	0.175	0.979	0.4074	1.7921	0.95
	308	30.30	0.0498	0.170	0.954	0.4009	1.7301	0.919
	318	30.30	0.0678	0.131	0.973	0.2917	1.9194	0.946
	328	29.41	0.0840	0.109	0.973	0.2624	1.9920	0.907
Fe_3_O_4_@BC	298	71.4	0.0700	0.1275	0.986	0.1786	1.425	0.998
	308	66.7	0.1049	0.0888	0.996	0.1419	1.435	0.988
	318	76.9	0.1048	0.0889	0.981	0.1291	1.348	0.984
	328	83.3	0.1188	0.0792	0.978	0.1205	1.233	0.974

The data exhibit excellent agreement with the Langmuir isotherm, with R^2^ values ranging from 0.954 to 0.979 for BC (Fig. 7c) and 0.987 to 0.996 for Fe_3_O_4_@BC (Fig. 7d). The calculated RL values fell within the range of 0.109–0.175 across the entire temperature study (298–328 K), indicating favorable adsorption conditions for both BC and Fe_3_O_4_@BC. The Freundlich isotherm also provides a reasonable fit, although the R^2^ values are lower than those obtained with the Langmuir model, as shown in Figs. 7a and b. the R^2^ values for BC ranges were 0.907–0.95 while it was 0.977- 0.998 for Fe_3_O_4_@BC.

Gibbs free energy ΔG serves as the thermodynamic criterion at constant pressure (P) and temperature (T) for determining whether a chemical process can occur or proceed. The enthalpy changes (ΔH) and entropy changes (ΔS) for the adsorption process of ACG2 on BC and Fe_3_O_4_@BC were derived from the plot of ln b versus 1/T^−1^, compiled in Table 4, and illustrated in Supplementary Figure S 8. The spontaneity of the reaction can be evaluated by examining the sign and magnitude of ΔG°. A negative ΔG° signifies the spontaneity of a chemical process. In designing any chemical process system, it is crucial to comprehend the changes expected during a chemical reaction. The rate and magnitude of these changes provide valuable insights for designing process equipment. An extensively negative value for ΔG° suggests the spontaneity of the adsorption process at a specific temperature (Figure S8b). With increasing temperature, the free energy values exhibit a positive trend, indicating a reduction in the spontaneity of the adsorption process at elevated temperatures. Positive ΔH° values denote the endothermic nature of the adsorption. The positive values of ΔS° indicate an increase in randomness at the solid/solution interface during the adsorption of ACG2 onto BC Fe_3_O_4_@BC.

**Table 4:** Thermodynamic parameters for ACG2 biosorption onto BC and Fe_3_O_4_@BC

Adsorbent	Temp. (K)	C_0_ (mg L^−1^)	ΔH° (kJ mol^−1^)	ΔS° (J mol^−1^ K)	ΔG° (kJ mol^−1^)
BC	298	24.3	27.208	118.635	− 35.326
	308	48.1	11.773	62.745	− 19.313
	318	72.4	11.248	59.490	− 18.906
	328	97.8	4.832	35.288	− 11.569
Fe_3_O_4_@BC	298	24.3	594.576	2073.635	− 617.349
	308	48.1	118.942	444.788	− 136.876
	318	72.4	72.433	279.165	− 88.702
	328	97.8	38.332	161.142	− 52.816

### Adsorption mechanism and comparison with other studies

The Fe_3_O_4_@BC composite exhibits a high surface-to-volume ratio characterized by the presence of pores, pits, holes, and surface defects, as indicated in Table 1. These structural features enabled the composite to adsorb the investigated ACG2 dye via physical and/or chemical mechanisms. The adsorption of dyes can occur within the interstitial spaces or defects among the carbon nanoclusters. Furthermore, additional physical adsorption mechanisms, including pore diffusion, van der Waals forces, π-π interactions, steric effects, and hydrophobic-hydrophobic interactions, may also facilitate dye uptake [68]. Furthermore, chemical adsorption pathways involving electron sharing between the dye molecules and the composite surface may occur. In this instance, an elevation in the solution's pH led to the neutralization of positive charges on Fe_3_O_4_@BC, along with an augmentation in surface negative charges which induced electrostatic repulsion between anionic dye molecules and the negatively charged Fe_3_O_4_@BC surfaces. This phenomenon resulted in a reduced adsorption potential of the Fe_3_O_4_@BC nanocomposite. Consequently, the optimal pH is identified as 1, a conclusion that is also supported by findings concerning the adsorption of Congo Red on cationic-amino modified banana leaves [57].

In recent years, magnetic biochar nanocomposites have been widely used in the field of dye removal. Algal-based magnetic biochar proved to be an efficient adsorbent for ACG2 adsorption. Compared with other studies, the maximum adsorption of similar dyes on other magnetic biochar at the same temperature was predicted through extrapolation of the curves. Biochar prepared from bamboo sawdust through a hydrothermal carbonization method shows a maximum ACG2 adsorption of 49.93 mg/g at 298 K [69], leading to a similar adsorption capacity in this research, which is 54.8 mg/g at 298 K. Therefore, algal-based magnetic biochar can be efficiently and eco-friendly used in dye wastewater treatment.

### Reusability of Fe_3_O_4_@BC composite

The ACG2-loaded Fe_3_O_4_@BC composites were regenerated via treatment with anhydrous ethanol, followed by filtration and drying at 373 K for 12 h. The recovered Fe_3_O_4_@BC composite was reused for five consecutive cycles for ACG2 adsorption. Results indicate that the percentage of ACG2 adsorption decreased from 96 to 83% over the course of five cycles (Supplementary Figure S9). The observed reduction in adsorption efficiency after regeneration can be attributed to weight loss during the adsorbent recovery process. Nevertheless, the observed loss is acceptable for the adsorption and recovery of ACG2.

## Conclusions

This study conducted a comprehensive investigation into the adsorption of Azocarmine G2 onto marine algal-based magnetic biochar (Fe_3_O_4_@BC), with a focus on various factors influencing the adsorption process. These factors include pH, adsorbent dosage, initial dye concentration, temperature effects, kinetics, isotherm modeling, adsorption mechanisms, and regeneration potential. The results indicate that the adsorption capacity (qe) of Fe_3_O_4_@BC for ACG2 is markedly influenced by the pH level. A maximum adsorption efficiency of 96% was observed at a low pH (approximately 1 and qe around 46 mg/g), which was attributed to enhanced electrostatic interactions between the positively charged surface of the biochar and the negatively charged dye molecules. An increase in the dosage of Fe_3_O_4_@BC improved the adsorption efficiency of ACG2 to 95.8%, peaking at an optimal point (2.5 g/L), beyond which only a slight increase in efficiency was noted, with the maximum qe reaching 71.3 mg/g. Kinetic analysis revealed that adsorption followed pseudo-second-order kinetics, implying a significant role of chemisorption in dye uptake. Isotherm studies demonstrated that both the Langmuir and Freundlich models could describe the adsorption behavior; however, the Langmuir model provided a superior fit, indicating monolayer coverage on a homogeneous surface. This finding suggests that magnetic biochar possesses a finite number of uniform sites for dye adsorption. Regeneration studies confirmed that magnetic biochar Fe_3_O_4_@BC could be effectively reused post-saturation, maintaining a significant portion of its initial adsorption capacity across multiple cycles. The algae biomass is sustainable and abundantly available in nature, and its utilization in biochar production, along with recyclability and regeneration, aligns with the SDG developmental goals, rendering this adsorbent highly suitable for large-scale wastewater treatment applications.

## Supplementary Information

Supplementary material 1.

## Data Availability

All authors ensure that all data, materials, software applications, and custom code support their published claims and comply with domain standards. The datasets used or analyzed during the current study are available from the corresponding author upon reasonable request.
