# Supplementary material for: Preparation and characterization of an algal-based magnetic biochar nanocomposite for the removal of azocarmine G2 dye from aqueous solutions
Source: BMC Chem. 2025 May 11;19(1):123. doi: 10.1186/s13065-025-01474-6 (PMC12067956; doi:10.1186/s13065-025-01474-6)
Supplement: Supplementary file 1 — Supplementary material 1. [file 13065_2025_1474_MOESM1_ESM.pdf]

Supplenetray figures for the Manuscript

Preparation and characterization of an algal-based magnetic biochar nanocomposite for the removal of azocarmine G2 dye from aqueous solutions

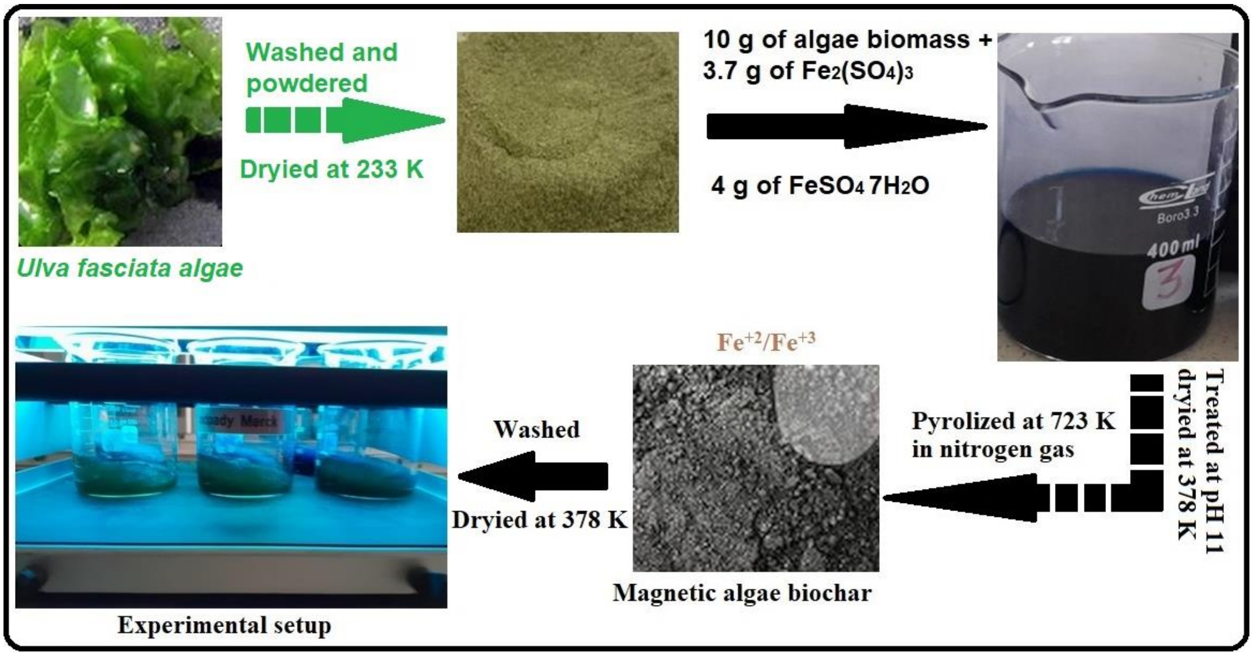

Fig S 1: Preparation of magnetic biochar Fe_3O_4@BC nanocomposites

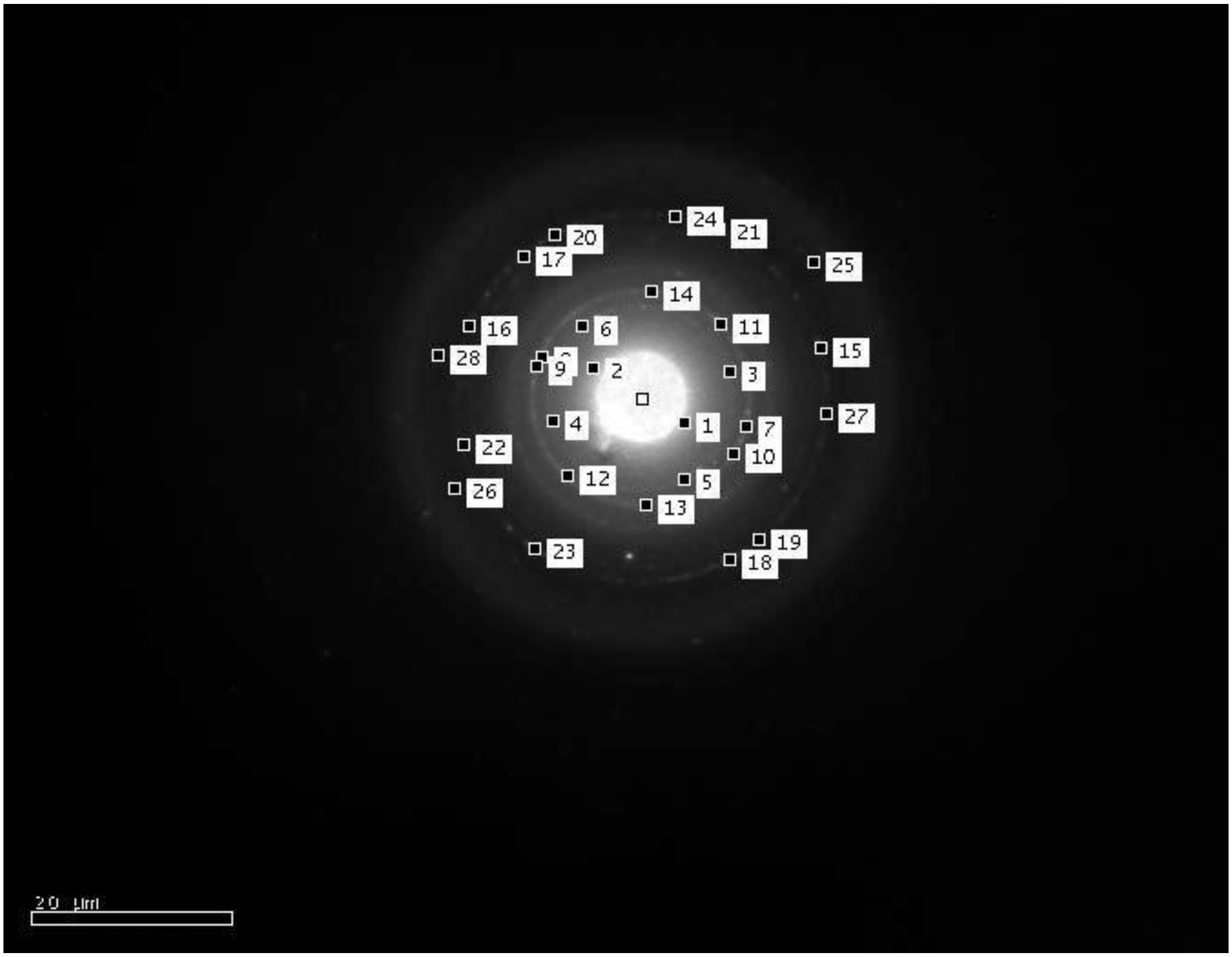

Figure S 2: Selective area electron diffraction (SAD) analysis of Fe_3O_4@BC

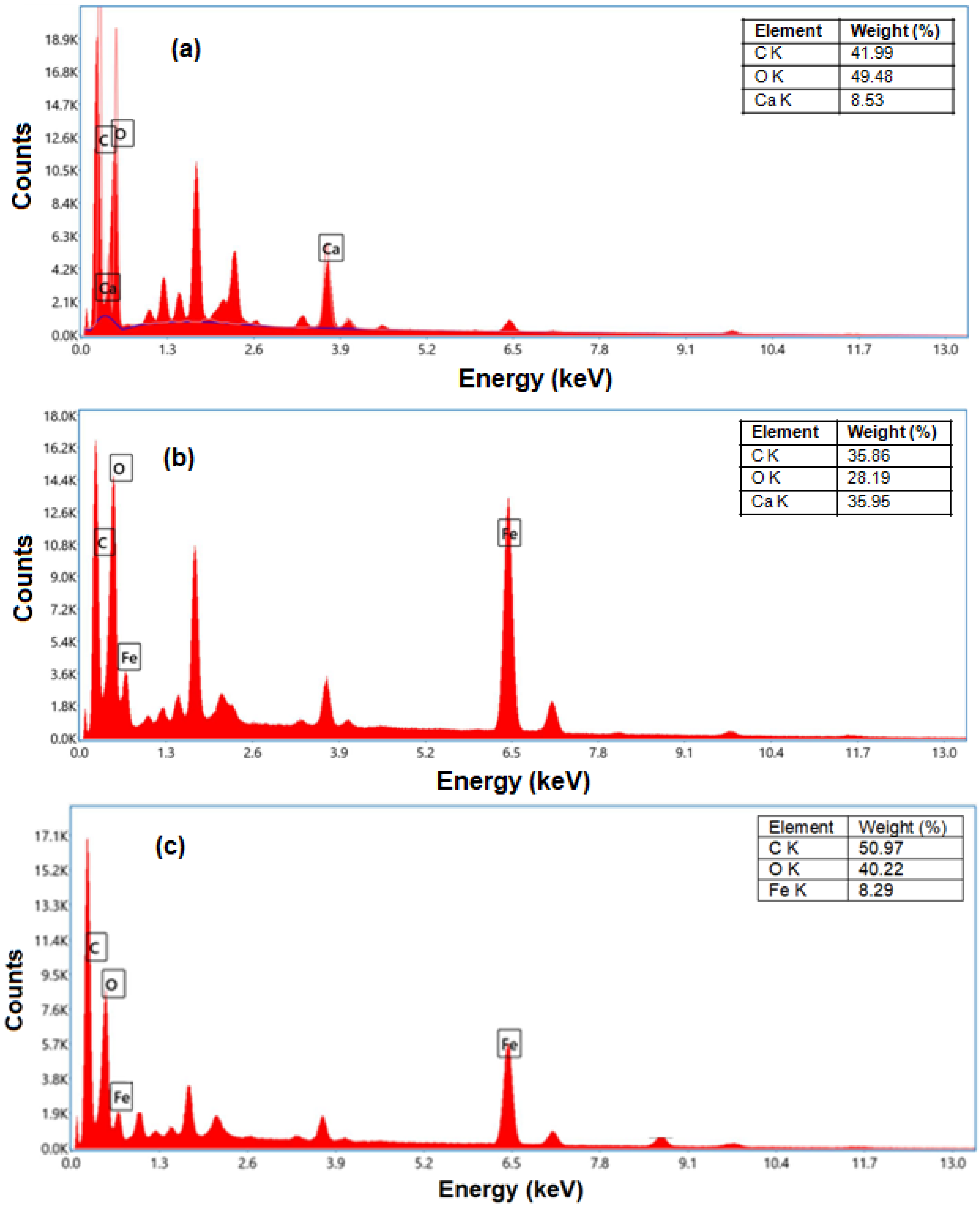

Figure S 3: EDX analysis of (a) BC, (b) Fe_3O_4@BC, and (c) Fe_3O_4@BC-ACG2

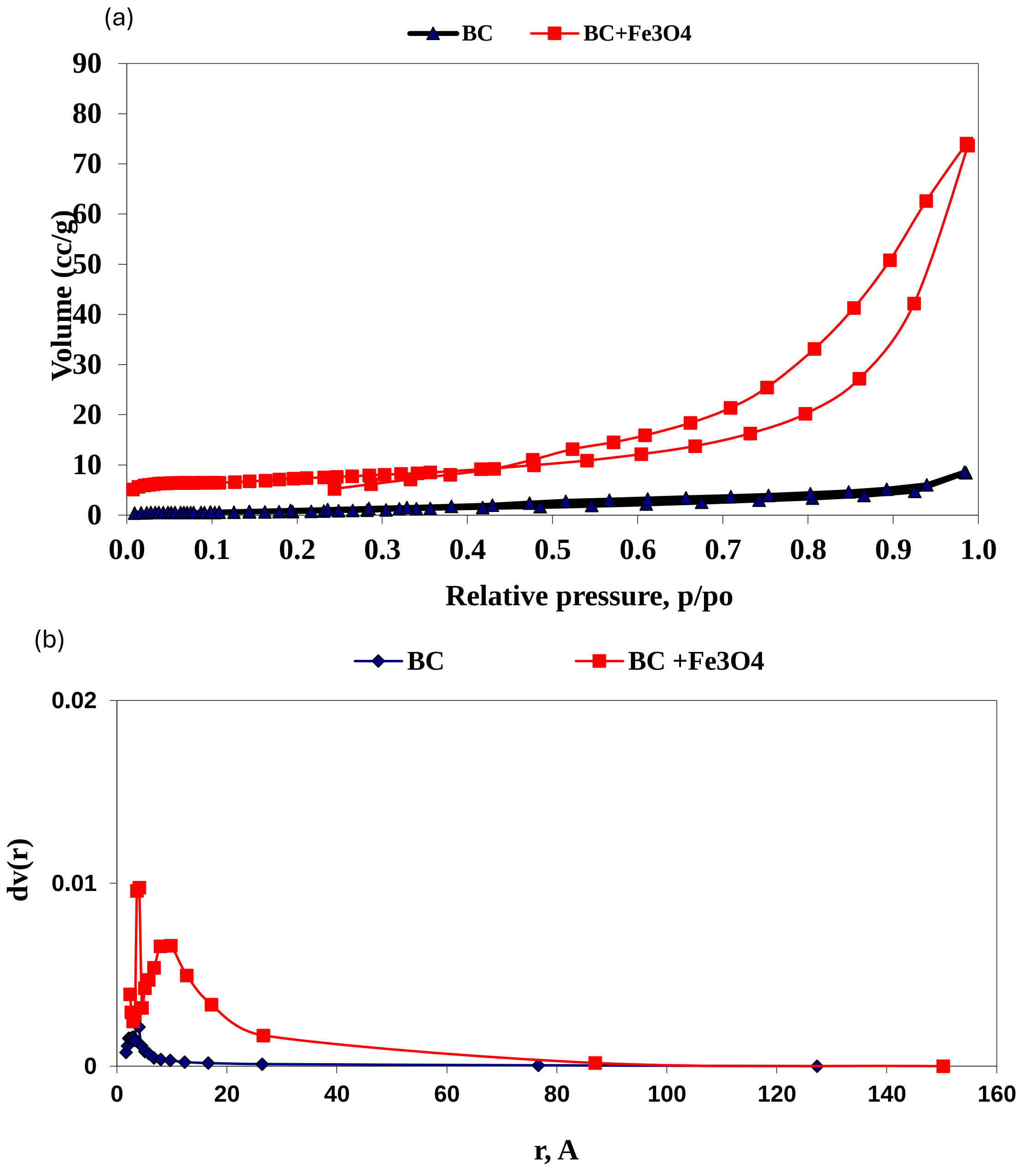

Figure S4: (a) N_2 adsorption–desorption isotherms of BC and Fe_3O_4@BC ; (b) BJH results obtained for BC and Fe_3O_4@BC

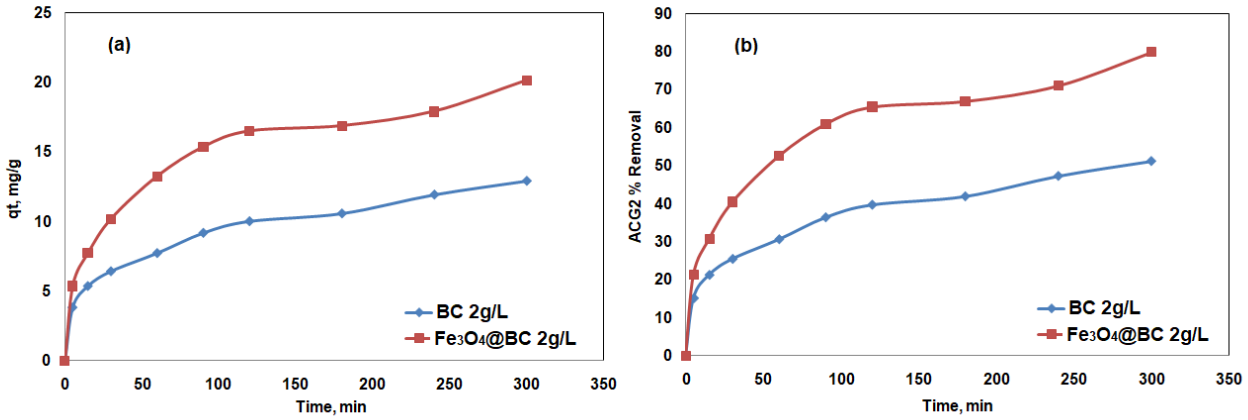

Figure S 5: Effect of time on adsorption of ACG2 on BC and Fe_3O_4@BC

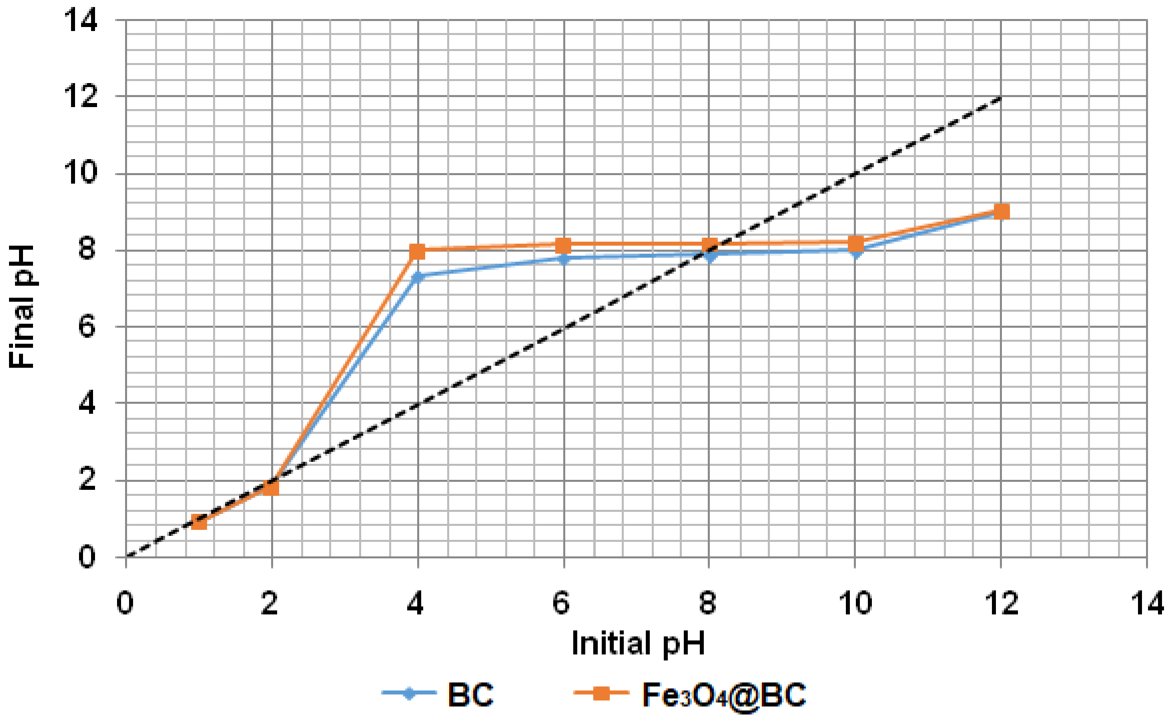

Figure S 6: pH PZC of adsorbents BC and Fe_3O_4@BC

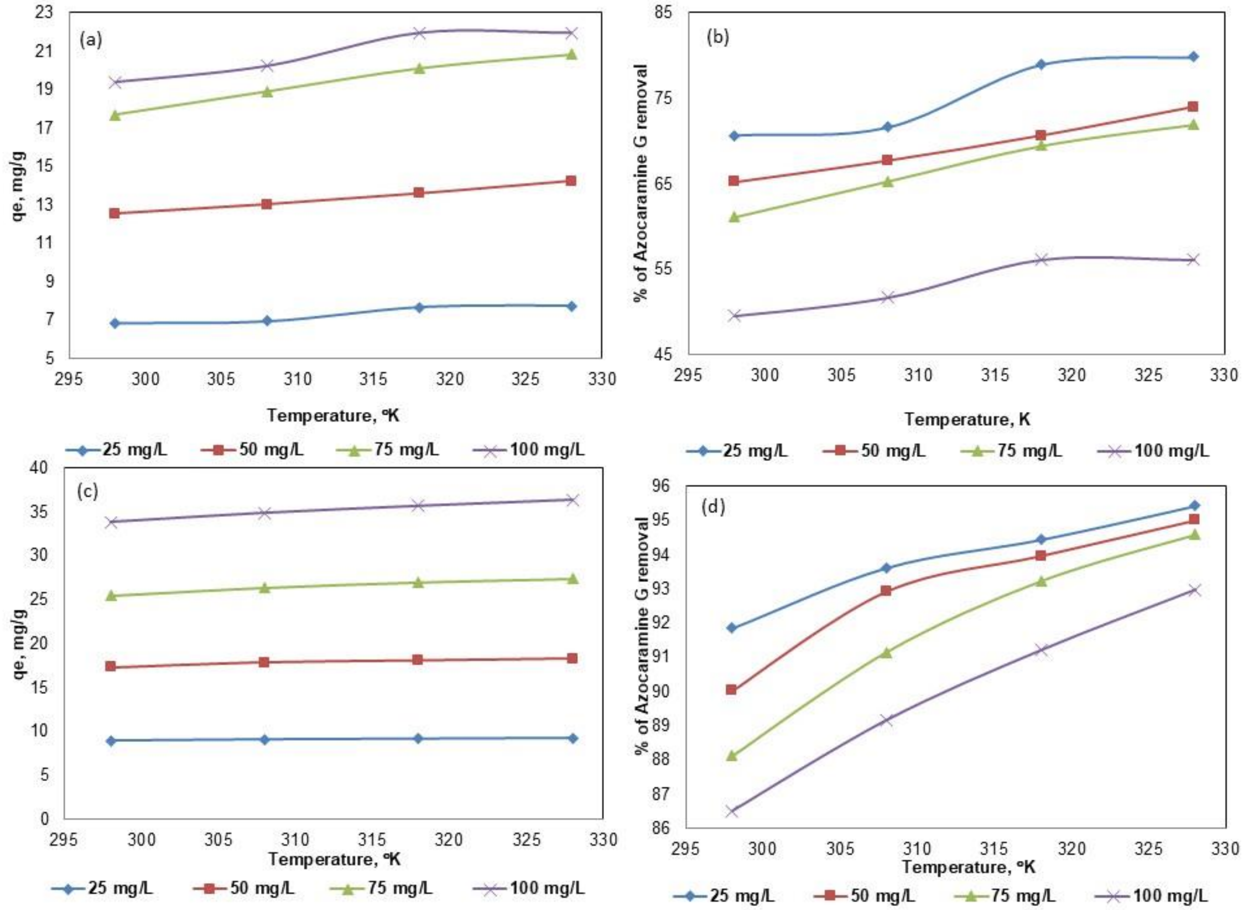

Figure S 7: Effect of temperature on adsorption of ACG2 on BC (a,b) and Fe_3O_4@BC (c,d)

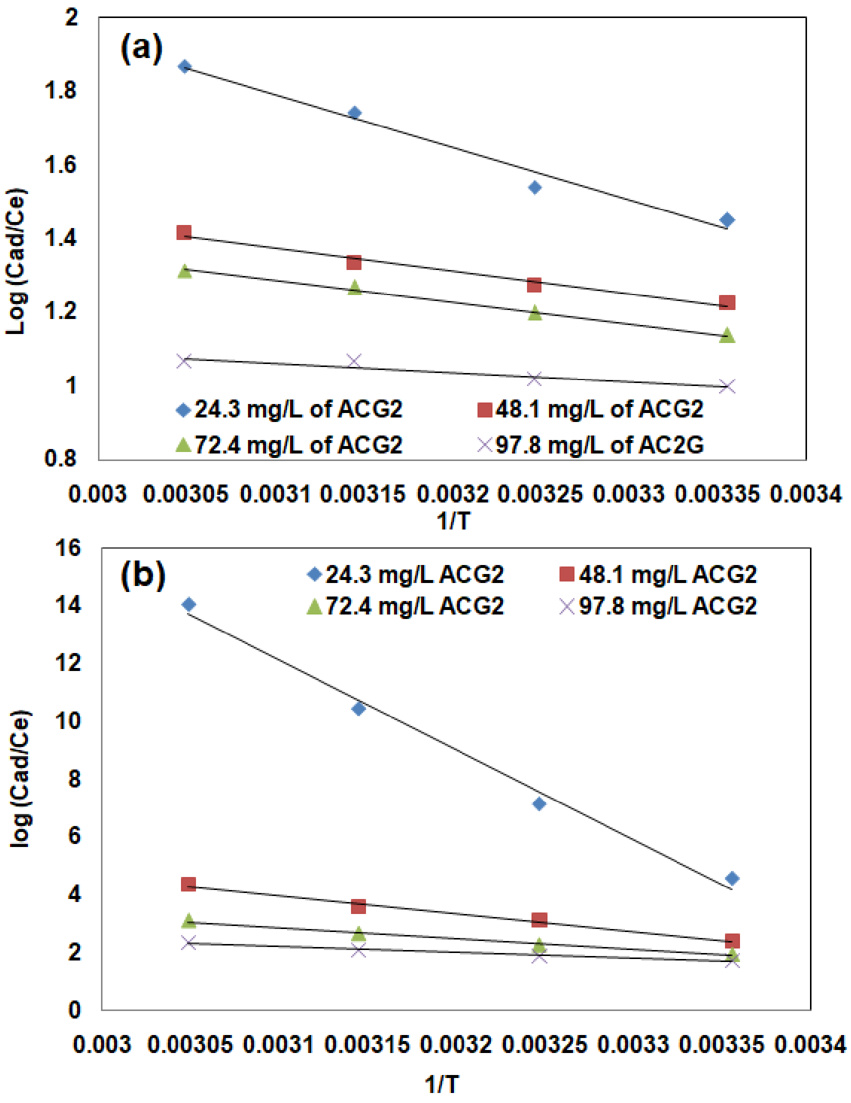

Figure S 8. Van't Hof plot of adsorption of ACG2 on BC (a) and Fe_3O_4@BC (b) adsorbents

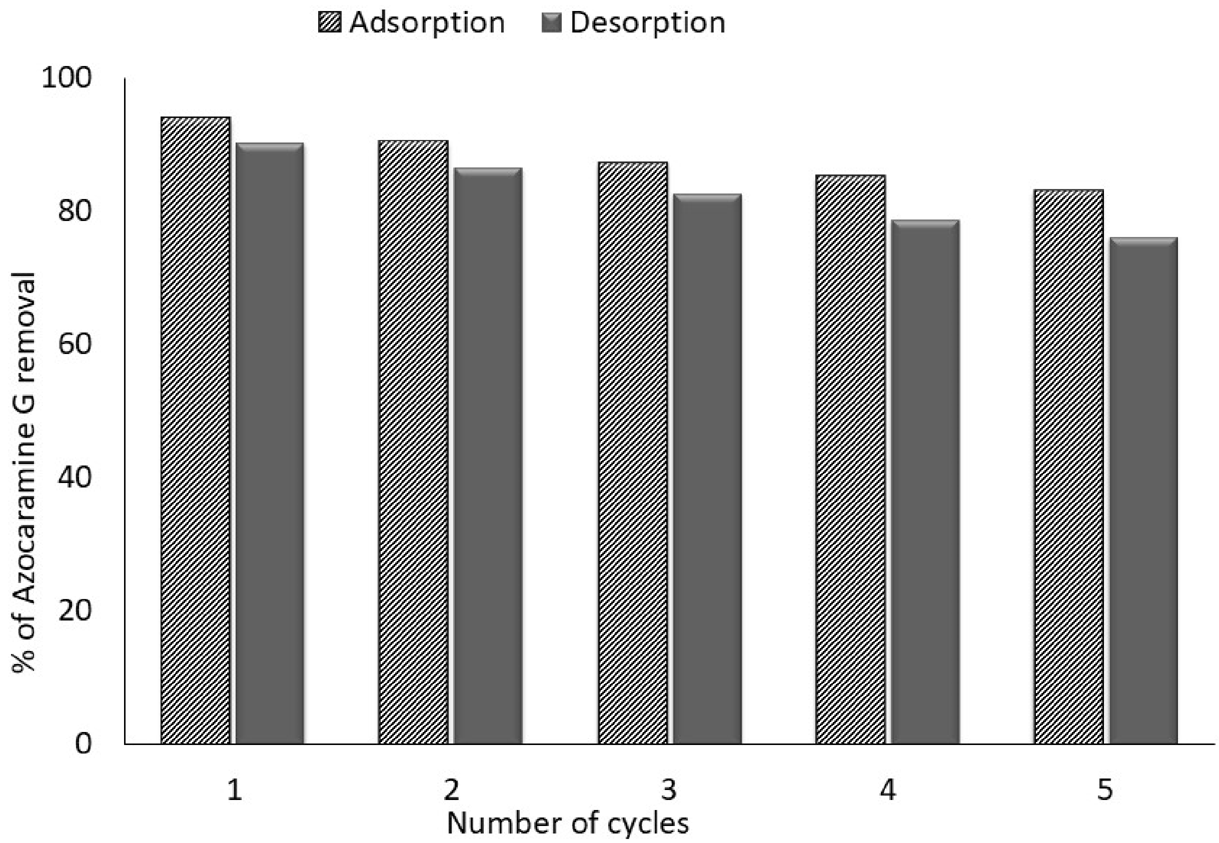

Figure S 9. Percentage adsorption of ACG2 at different reusability cycles Fe_3O_4@BC
